# Pasteurization Preserves IL-8 in Human Milk

**DOI:** 10.3389/fped.2018.00281

**Published:** 2018-10-10

**Authors:** Marilyn V. Giorgi, Champa N. Codipilly, Debra Potak, Howard S. Heiman, Richard J. Schanler

**Affiliations:** Lilling Family Neonatal Research Lab, Division of Neonatal-Perinatal Medicine, Cohen Children's Medical Center, Zucker School of Medicine at Hofstra, Northwell, Feinstein Institute for Medical Research, New York, NY, United States

**Keywords:** human milk, premature infants, donor human milk, pasteurized donor human milk, cytokines

## Abstract

**Background:** Pasteurized donor human milk is an alternative feeding when mothers' own milk is not available for premature infants. The effects of pasteurization on the host defense properties of human milk are unclear. We investigated the effects of Holder pasteurization on concentrations of anti-inflammatory and pro-inflammatory cytokines in human milk.

**Objective:** To compare concentrations of anti-inflammatory and pro-inflammatory cytokines before and after pasteurization of donor human milk.

**Study Design:** A single milk sample was obtained from each of 24 mothers of premature infants in the neonatal intensive care unit by electric breast pump and was stored at −80°C. At the time of pasteurization, milk samples were thawed and divided into two aliquots. The first aliquot was re-stored at −80°C and the second aliquot was heat-treated at 62.5°C for 30 min and then re-stored at −80°C. At the time of batch cytokine analyses samples were thawed rapidly.

**Results:** Most cytokine concentrations declined following pasteurization. The most prevalent cytokine, IL-8, was preserved (89%) following pasteurization. There were no relationships between gestational age, postnatal age of milk collection, duration of milk storage, and the concentrations cytokines.

**Conclusion:** In contrast to most cytokines after pasteurization, IL-8 is preserved or liberated from another compartment. The maintenance of IL-8 in human milk after pasteurization and the loss of anti-inflammatory cytokines following pasteurization, suggests that the effects of inflammatory activity in pasteurized human milk should be evaluated. These data may account, in part, for the lesser protective effect on the host of pasteurized donor human milk compared with mother's own milk.

## Introduction

There is strong evidence to support feeding premature infants their mother's own milk (1–3). Not all mothers of premature infants, however, are able to supply sufficient milk to meet the needs of their infants throughout the NICU stay. When mother's own milk is not available, most clinicians recommend pasteurized donor human milk (DHM) as the second choice for feeding premature infants (4). There are advantages of using DHM as well as concerns limiting its use. When compared with preterm formula, DHM is associated with lower rates of necrotizing enterocolitis and lower mortality (4–6).

Pasteurized donor milk may not confer the same protective effect as mothers' own milk in the feeding of premature infants (7, 8). Donor milk is dissimilar to mother's own milk because it usually is obtained later in lactation when the contents of certain nutrients (protein, sodium) are lower and nutrient losses may have occurred from the collection and storage processes (lipid). Moreover, specific component concentrations may be affected by the heat-treatment process (7, 8). Holder pasteurization, heating milk to 62.5°C for 30 min, the usual method for processing donor human milk, is associated with substantial losses of immune components, including lactoferrin, secretory IgA, and lysozyme, and these losses are variable (9–11).

The cytokines in human milk (usually IL-1β, IL-2, IL-4, IL-5, IL-6, IL-8, IL-10, IL-12, EGF, TGF-α, TGF-β, TNF-α, and IFN-γ) are believed to provide passive immunity to the neonate (12–15). These cytokines are believed affect the maturation of the developing human intestine (16). As there are scant data on the effect of heat treatment on total cytokine concentrations in donor human milk, the objective of this study was to assess the effect of human milk pasteurization on its cytokine concentrations.

## Methods

A single milk sample of 50 mL from a complete collection of one breast was obtained from 24 mothers of premature infants in the NICU using an electric breast pump. Mothers were free of medical illnesses and not receiving medications, including antibiotics. Milk samples were collected fresh and stored at −80°C in sterile polypropylene containers until studied. Samples were thawed at room temperature and divided into two equal parts. The first part was divided into 2 mL aliquots and re-stored at −80°C until analyzed and the second part was heat-treated in a shaking water bath at 62.5°C for 30 min. A thermometer was placed in a centrally located non-study milk sample to ensure all study samples were maintained at 62.5°C. Subsequently, 2 mL aliquots of the pasteurized milk samples were re-stored at −80°C until analyzed.

Just prior to analyses, milk samples were thawed rapidly and centrifuged at 3000 rpm for 30 min to separate lipid and aqueous layers. Cytokine concentrations in aqueous milk samples were determined by flow cytometry (BD Facscaliber Flow Cytometer, San Diego, CA). Human Inflammation and Human Th1/Th2 cytokine kits (BD Cytometric Bead Array Analysis, San Diego, CA), following manufacturers protocols, were used for individual cytokine analyses. The use of both kits allowed an analysis of 10 cytokines: IL-1β, IL-2, IL-4, IL-5, IL-6, IL-8, IL-10, IL-12p70, TNF-α, IFN-γ. IL-10, and TNF-α measured by both kits were the same suggesting no interassay variation.

Paired samples (before- and after-pasteurization) were evaluated for IL-1β, IL-2, IL-4, IL-5, IL-6, IL-8, IL-10, TNF-α, IL-12p70, and IFN-γ concentrations. The adequacy of pasteurization was confirmed by comparing the concentrations of alkaline phosphatase and lipase in before and after treatment samples.

Sample size was chosen to detect a difference of one standard deviation from the mean. We chose a significance of *p* = 0.005 due to multiple comparisons and a power of 0.80. Pearson correlation coefficients were used to compare relationships between variables. Before- and after-pasteurization results were analyzed by paired *t*-test. The data were standardized using z-scores, then the changes of IL-8 were compared to the other nine cytokines measured using a RMANOVA model.

The study was approved by the Institutional Review Board of the North Shore Long Island Jewish Health System (now known as Northwell Health). Written informed consent was obtained from all mothers.

## Results

Milk samples, collected at 4–54 days postpartum from mothers delivering infants between 27 and 36 weeks gestation, were stored at −80°C for 8–157 days prior to analyses. There were no relationships between gestational age, postnatal age of milk collection, duration of milk storage, and the concentrations of cytokines in milk (*p* > 0.05). The completeness of pasteurization was confirmed by measuring milk alkaline phosphatase (mean 1946 and 9 U/L) and lipase (mean 48.8 and < 3 U/L) before and after treatment, respectively, *p* < 0.005.

**Figure 1:**
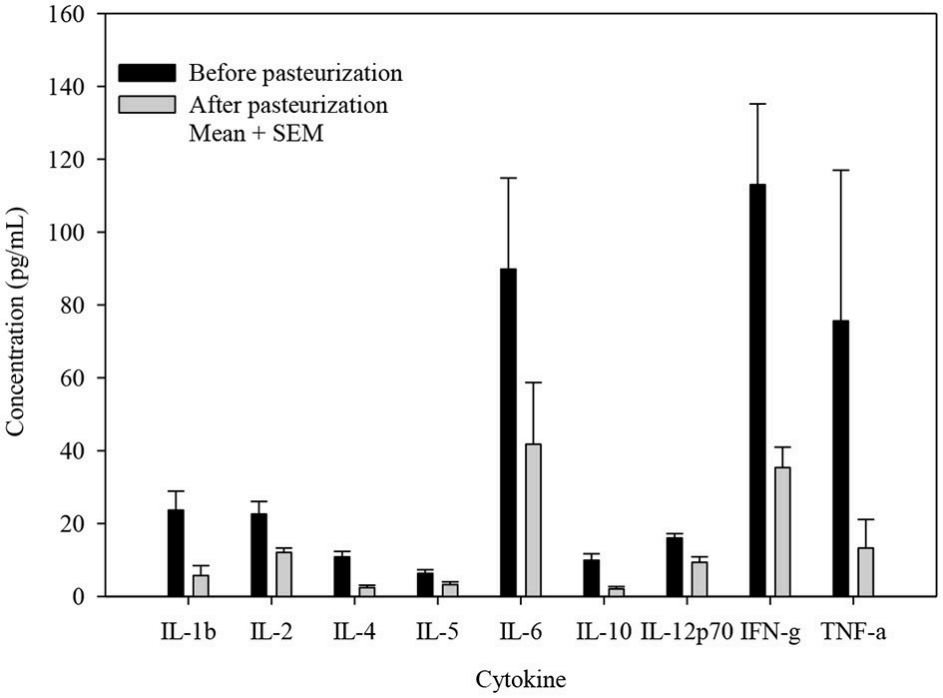
Cytokine concentrations before and after pasteurization. The concentration of cytokines of 24 milk samples measured in duplicates in thawed human milk before and after pasteurization. All cytokine concentrations decreased significantly, *p* ≤ 0.005.

**Figure 2:**
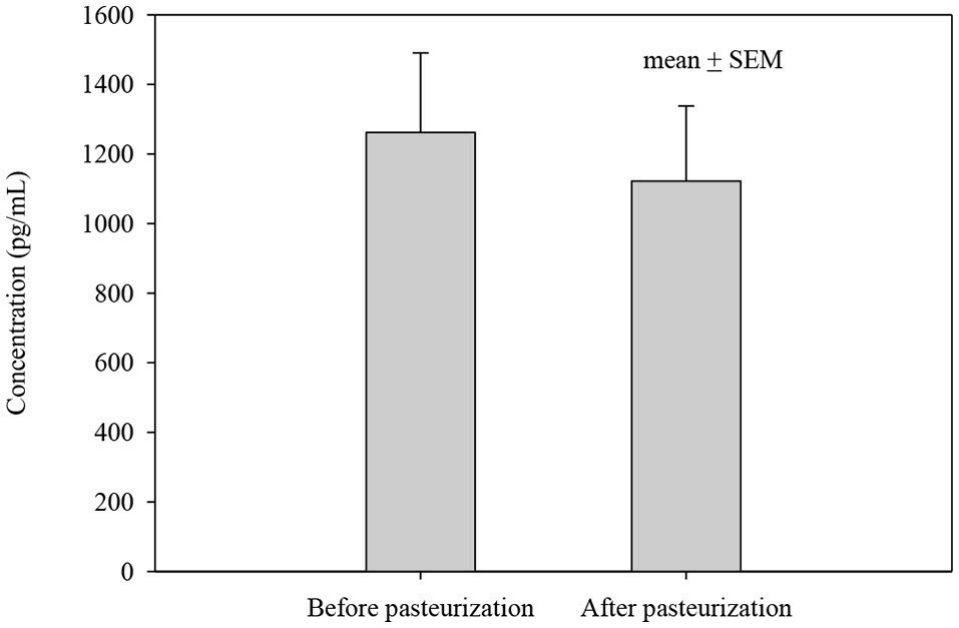
IL-8 concentration before and after pasteurization. The concentration of IL-8 of 24 milk samples measured in duplicates in thawed milk. The concentration after pasteurization was not changed significantly, *p* = 0.543.

**Figure 3:**
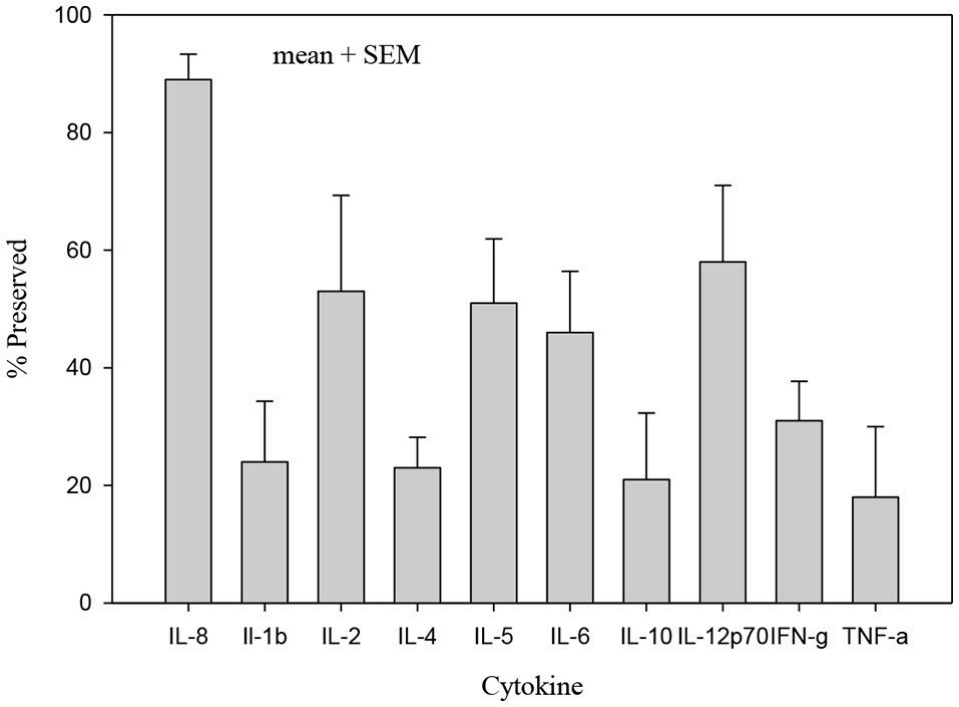
Percentage of cytokine preserved after pasteurization. Only IL-8 concentration in heat treated human milk was preserved (89%) compared with other cytokines which were significantly less preserved after heat treatment, *p* < 0.005.

The concentrations of all cytokines declined following pasteurization (Figures 1, 2). There was variable preservation (18–58%) of cytokines following pasteurization. The most abundant cytokine, IL-8, was preserved (89%) after pasteurization compared to other cytokines (Figure 3).

## Discussion

Mother's milk provides important protection to the premature infant who has a developmentally delayed immune system (17). As not all mothers of premature infants are able to provide 100% of their infants' needs, donor human milk has emerged as an alternative feeding strategy. As a result there is an increased interest in donated human milk and a marked increase in donor milk banks in the US. Donor human milk is heat treated for the prevention of microbial transmission. There are concerns that because of its heat treatment donor human milk may not provide the same protective benefit as mother's own milk (2, 3, 6, 18). Indeed, when supplemented with bovine milk products, pasteurized human milk is associated with more infectious morbidity than non-heat treated milk, suggesting that there may be some loss of immune properties once human milk is heat-processed (4, 19).

Among the immune components in human milk are a variety of cytokines, whose concentrations vary with lactation stage, from colostrum to mature milk, between foremilk and hindmilk, between mothers delivering at term or prematurely, and with maternal medical conditions (20, 21). We found using a reproducible common laboratory assay that pasteurized human milk contained both pro- and anti- inflammatory cytokines (20, 22–24). We found a generalized reduction in cytokine concentrations after pasteurization. The pro-inflammatory cytokine IL-8 was the most preserved (89%) cytokine after pasteurization. Despite the measured declines in anti-inflammatory cytokines after pasteurization, the retention rates of 18–58% suggest that donor milk still provides potential protection to the recipient infant.

Our data qualitatively reinforce other reports of the preservation of IL-8 after pasteurization (15). We speculate that the preservation of IL-8 in heat-treated milk may be a concern for the premature infant who is susceptible to a variety of inflammatory conditions. IL-8, a major mediator of inflammatory responses and a chemoattractant, is found in leukocytes and endothelial cells. Its preservation may be a result of a strong tertiary structure (25). The other two pro-inflammatory cytokines preserved at a higher levels are IL-2 (53%) and IL-12p70 (58%), probably also due to their protein structure.

The imbalance favoring pro-inflammatory cytokines in pasteurized milk potentially could explain the lack of consistent short-term benefits of this milk when compared with mother's own milk (6, 18, 26). There are inconsistent beneficial outcomes of inflammation-related diseases in premature infants receiving donor human milk: necrotizing enterocolitis, bronchopulmonary dysplasia, and retinopathy of prematurity (6, 26–29). Moreover, long-term studies fail to demonstrate better outcomes of pasteurized donor milk-fed infants (30). Nevertheless, when compared with preterm formula, short-term benefits of pasteurized donor milk are noted (31).

Our data might be used to question the process of pasteurization, classic Holder pasteurization, as short-time high temperature pasteurization methods have been associated with less protein degradation (32). Indeed, the high temperature treatment reported greater retention of bioactive factors in human milk (IgA, alkaline phosphatase, bile salt-stimulated lipase) with similar antibacterial efficacy when compared with Holder pasteurization. The expense and practicality of the high temperature method needs further evaluation to encourage its use.

Thus, we report preservation of pro-inflammatory cytokines after pasteurization of human milk suggesting that heat treatment may have significantly different effects on the premature infant with a developing immune system.

## Ethics statement

This study was carried out in accordance with the recommendations of the Human Subjects Review Committee of the North Shore Long Island Jewish Health System (now known as Northwell Health). The protocol was approved by the Institutional Review Board of the North Shore Long Island Jewish Health System (now known as Northwell Health). All subjects gave written informed consent in accordance with the Declaration of Helsinki.

## Author contributions

All authors listed have made a substantial, direct and intellectual contribution to the work, and approved it for publication.

### Conflict of interest statement

The authors declare that the research was conducted in the absence of any commercial or financial relationships that could be construed as a potential conflict of interest.
